# Entrapment of Ciliates at the Water-Air Interface

**DOI:** 10.1371/journal.pone.0075238

**Published:** 2013-10-10

**Authors:** Jonathan Ferracci, Hironori Ueno, Keiko Numayama-Tsuruta, Yohsuke Imai, Takami Yamaguchi, Takuji Ishikawa

**Affiliations:** 1 Department of Engineering, Tohoku University, Sendai, Japan; 2 Faculty of education, Aichi University of Education, Aichi, Japan; 3 Department of Biomedical Engineering, Tohoku University, Sendai, Japan; 4 Department of Bioengineering and Robotics, Tohoku University, Sendai, Japan; University of Hull, United Kingdom

## Abstract

The importance of water-air interfaces (WAI) on microorganism activities has been recognized by many researchers. In this paper, we report a novel phenomenon: the entrapment of ciliates *Tetrahymena* at the WAI. We first characterized the behavior of cells at the interface and showed that the cells' swimming velocity was considerably reduced at the WAI. To verify the possible causes of the entrapment, we investigated the effects of positive chemotaxis for oxygen, negative geotaxis and surface properties. Even though the taxes were still effective, the entrapment phenomenon was not dependent on the physiological conditions, but was instead affected by the physical properties at the interface. This knowledge is useful for a better understanding of the physiology of microorganisms at interfaces in nature and in industry.

## Introduction

Information on near-wall behaviors of microorganisms is important in understanding various phenomena such as biofilm formation, corrosion, and the dynamics of intestinal flora. Therefore, these behaviors have been investigated intensively. For example, it is well known that bacteria swim with a circular trajectory when close to solid surfaces [1]–[3]. Other studies reported the accumulation of bacteria near a wall [4], or the motion of spermatozoa that form a grid of circles of 5–6 cells when swimming close to a solid wall [5]. Such observations led to theoretical studies of the behavior of squirmers close to a solid surface [6], [7]. Recently Lauga & Powers [8] reviewed basic theories of near-wall behaviors of microorganisms. They made the distinction between two types of swimmers: pullers, such as *Chlamydomonas*, and pushers, such as bacteria, and showed that a pusher is attracted by a solid surface, whereas a puller is always orientated away from a solid surface.

On the other hand the fluid mechanics of swimming microorganisms at water-air interface (WAI) is still largely unknown, even though the importance of WAI on microorganism activities has been recognized by many researchers. Kristiansen et al. [9], for example, highlighted the importance of WAI in the growth rate of *Tetrahymena*. They clarified that the ratio of the surface area to depth of the culture vessel considerably affected the survival rate of *Tetrahymena*. Schafer et al. [10] studied the difference in accumulation of bacteria at the WAI with regard to their hydrophobicity and charge. Additionally, several groups have investigated the effect of the surfactant on the interaction of bacteria with hydrophobic interfaces, such as the water-gas interface. They have shown that the addition of surfactant prevents biofilm formation at any kind of interface [11], [12]. Recently, Marcos et al. [13] showed that the presence of microorganisms in the bulk below the WAI greatly affected the scattering of light in marine environments, which in turn, may affect organisms in the upper ocean. These studies clearly illustrated the strong interaction between the WAI and microorganisms. In terms of fluid mechanics, however, the behavior of swimming microorganisms at the WAI has not been fully clarified to date.

**Figure 1:**
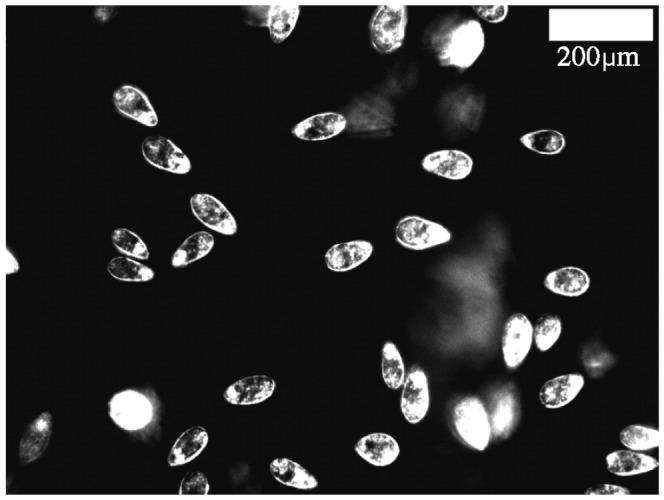
Image of *Tetrahymena* cells trapped and swimming at a water-air interface (WAI).

In this paper, we report for the first time the entrapment of swimming ciliates at a WAI. We observed the entrapment for both *Tetrahymena thermophila* and *Volvox carteri*, which have been widely used in biological studies as model ciliates. When we put a droplet containing *Tetrahymena* or *Volvox* on a glass slide, we saw cells trapped at the WAI for tens of seconds, as shown in Fig. 1 and in Movies S1 and S2. In the case of *Volvox*, similar entrapment was reported even in the vicinity of a solid wall, where cells danced stably [14]. The stability is mainly attributed to the bottom-heaviness of the cells, which tends to align the cells vertically upward on average. In contrast, the behavior of *Tetrahymena* was very different for the WAI compared with water-solid interfaces. *Tetrahymena* tend to swim away from a solid wall, as shown in Movie S3, but become trapped at the WAI (cf. movie S1). We see from Movie S1 that the *Tetrahymena* trapped at the WAI swam very slowly compared with those inside the medium. The decrease (increase) in the swimming velocity occurred instantaneously when a cell was trapped at (escaped from) the interface. The trapped cells also generated strong mixing flow around the cells, as seen in the Movie S4. Therefore the mass transport in the medium as well as the nutrient uptake by the cells is likely to be affected by entrapment. In this study, we concentrated our attention on *Tetrahymena*, to determine the physical and/or biological mechanism behind the entrapment phenomenon.

Herein, we show that the mechanism of entrapment of *Tetrahymena* at the WAI can be explained by interfacial physics. In our study, we first characterized the entrapment phenomena experimentally and then investigated the effects of aerotaxis, geotaxis and surface physical properties, to verify the possible causes of the entrapment.

## Results

### Characterization of the entrapment

**Figure 2:**
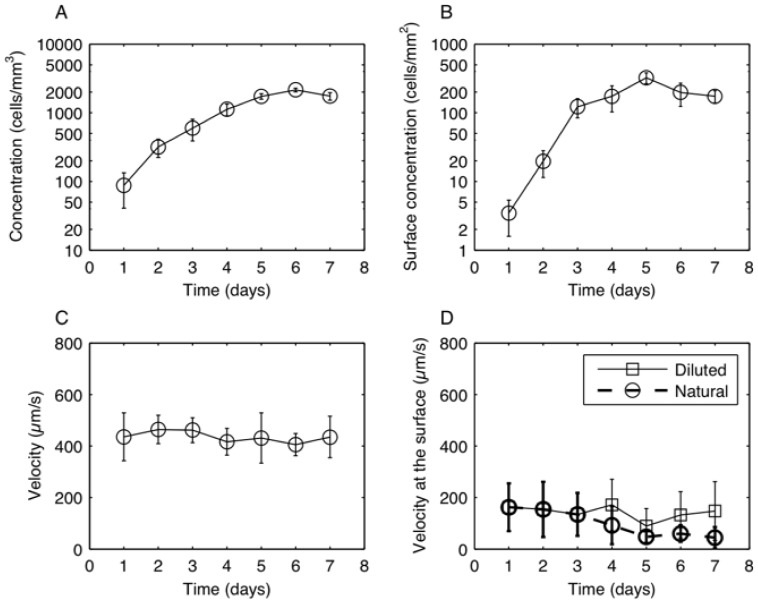
Characterization of cells' behavior away from interfaces and at the WAI. This was done for six different inocula over a period of 7 days. The error bars indicate the standard deviation. (A) Growth curve of *T. thermophila*. (B) Change in the surface concentration of *T. thermophila*. (C) Change in the swimming velocity away from any interfaces. (D) Change in the swimming velocity at the WAI in a diluted or natural suspension.

We first measured the change in cell concentration in the culture and surface concentration at the WAI for 7 days. The changes in concentration can be respectively seen in Fig. 2(A) and (B). The strong correlation between the number density of cells at the interface and inside the medium can be explained statistically. The probability of cells colliding at the interface increases with the number of cells existing inside the medium. The number of cells at the interface was limited due to a two-dimensional (2-D) geometry, which could explain why the plateau was reached first at the interface.

The main feature of entrapment was that the swimming velocity of the cells at the interface decreased significantly, compared with that inside the fluid. The change in the velocity at the interface was instantaneous; i.e., the cells suddenly decreased their swimming velocity when they were trapped at the interface, whereas cells suddenly increased their swimming velocity when they escaped from the interface (cf. Movie S1). We thus compared the swimming velocity at the interface and inside the fluid.

Figure 2(C) shows the change in the swimming velocity inside the medium as a function of time over the course of 7 days. We observed that the velocity of the cells away from the interface did not vary considerably over the 7-day period, and that the value was 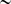
450 µm/s. At the interface, the swimming velocity decreased significantly, as shown in Fig. 2(D). In the natural culture fluid, the swimming velocity at the interface decreased to 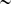
50 µm/s from the 5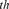 day. This is partly because the number density of cells at the interface became too large, and the swimming was obstructed by the other cells. To eliminate the cell-cell interaction effect, we also measured the swimming velocity at the interface with a diluted suspension. The diluted suspension was prepared by adding only the medium of the same day, where the suspended cells were removed by centrifugation, to a natural suspension. Figure 2(D) shows that the swimming velocity at the WAI was different for the natural and the diluted suspensions. In the diluted suspension, the cells swam with a velocity of 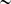
150 µm/s over a 7-day period. This confirmed that only the comparison of the swimming velocity at the interface in a diluted suspension and that inside the medium was relevant for observing the effect of the interface. The cells at the interface swam at 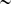
1/3 the velocity of those inside the medium. This indicated the presence of surface forces that hindered the swimming of the cells, or a modification of the biological function of the cells, making them swim more slowly. To understand the entrapment mechanism and the change in behavior at the interface, we examined the mechanisms in detail, as explained below.

### Effect of taxes on entrapment

There could be various reasons for why *Tetrahymena* tended to stay at the interface. It is known that *Tetrahymena* show both aerotaxis and geotaxis [15], [16]. Thus, cells naturally tend to swim upwards, i.e. in the direction of positive gradient oxygen concentration as well as in the negative gravitational direction. These biological responses may play a role in the entrapment of cells at the interface. Another possible cause is hydrodynamic forces at the interface. The deformation of the surface, as well as the stress-free conditions of a flat WAI could alter the hydrodynamics around the cells [17]. The investigation of these possible causes was conducted experimentally on the 5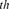 day after inoculum. Measurements were made for six different samples, in which the surface velocity was calculated for the diluted suspension.

**Figure 3:**
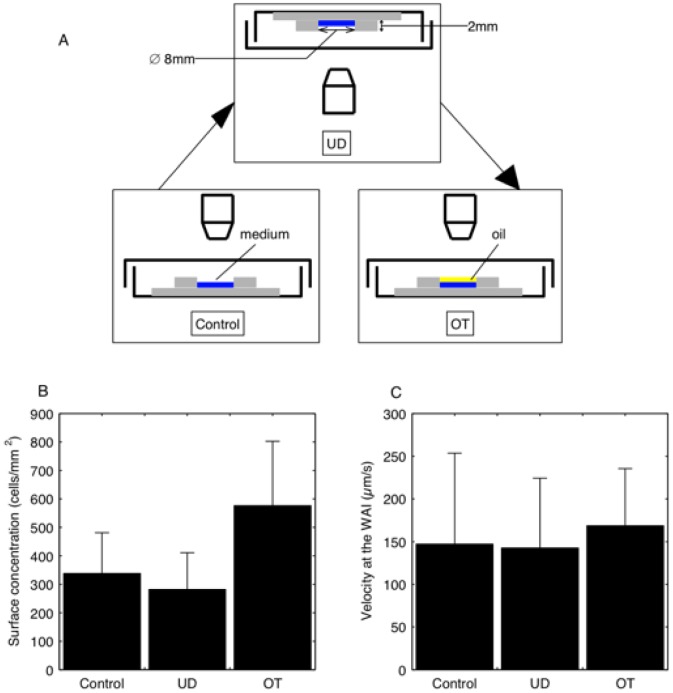
Behavior of cells under three different physiological conditions at day 5 (for six different samples): (A) set-up, (B) surface concentration, and (C) swimming velocity.

To begin, we considered two possible biological causes: positive aerotaxis and negative geotaxis. This allowed us to determine if entrapment was a possible cause, or if a strong attraction phenomenon caused the cells to stay at the interface and to swim in two dimensions as if trapped. To evaluate the effect of these two taxes, three successive experiments were conducted, as shown in Fig. 3(A). We first observed the cells' behavior in the control setting, then turned the pool upside-down to see the effect of geotaxis, and finally returned it to its original position. Mineral oil was then added to the top to see the effect of aerotaxis. The upside-down condition was observed with an inverted microscope (Olympus IX71, Japan). An 8-mm-diameter pool, instead of the 25-mm-diameter pool, was used to keep the upside down condition stable. This set of experiments was performed on day 5. The swimming velocity of the cells was measured in the diluted suspension to avoid cell-cell interactions.

Figures 3(B) and (C) show the values of the surface concentration and the swimming velocity of the cells at the WAI for the three conditions. The swimming velocity of the cells at the WAI was in the range of 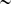
100–200 µm/s, which was significantly lower than the value of 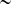
450 µm/s inside the medium. The surface concentration was high in all three cases. These results indicated that the effects of positive aerotaxis and negative geotaxis were small in the entrapment phenomenon, though they may affect the orientational change of cells inside the medium. The results with the upside-down condition also indicated that the effect of sedimentation due to gravity was negligible in the present system. Because no obvious change in the entrapment phenomenon was observed in the three experiments, we turned our attention to the possible effect of physical surface properties on cell entrapment at the interface.

### Effect of a surfactant on the behavior at the WAI

**Figure 4:**
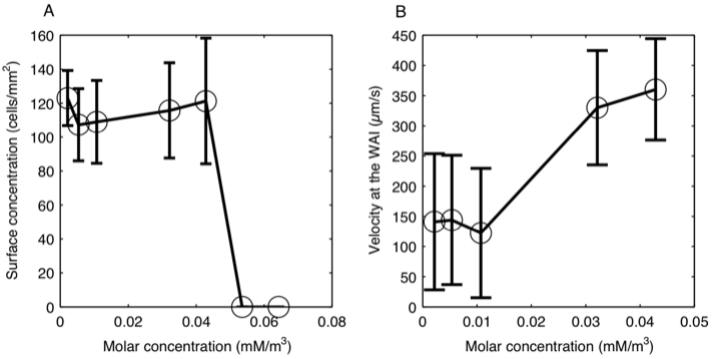
Effect of surfactant (Tween 20) concentration on the entrapment for day 5: (A) change in the surface concentration; note the presence of a step at 0.05 mM/m^3^, and (B) change in the swimming velocity at the WAI.

To study the effect of surface properties, we added a surfactant to the medium and observed the entrapment phenomenon under this condition. We used Tween 20 (KWH506, Wako, Japan) as a surfactant, which allowed modification of the surface properties with little effect on the biology of the cells. Figure 4(A) shows that the surface concentration of cells at the WAI did not change much when the surfactant concentration was below 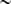
0.05 mM/m^3^. When the surfactant concentration was equal or higher than 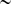
0.05 mM/m^3^, however, the number of cells in the vicinity of the WAI drastically dropped, as they were able to easily swim away from the interface without any significant change in velocity. Figure 4(B) shows the velocity of the cells at the WAI when surfactant was included, that is, for a value inferior to 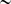
0.05 mM/m^3^. When the surfactant concentration increased from nearly 0 to 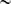
0.04 mM/m^3^, the swimming velocity of the cells doubled from 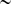
150 µm/s to 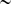
350 µm/s even though we did not observe any change in the swimming velocity inside the medium as we added the surfactant. We note that the critical micelle concentration (CMC) of Tween 20 in pure water is 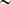
0.06 mM/m^3^ [18]. The cells' behavior thus changed dramatically, when the surfactant concentration reached the CMC condition.

The behavior of cells at the WAI with surfactant concentrations greater than 0.05 mM/m^3^ was similar to that at a solid-liquid interface: i.e., the cells were not trapped. In understanding this fact, it is important to remember that the absorbed surfactant modifies the hydrodynamic boundary conditions at the WAI: bulk stresses are coupled to surface stresses that arise from surface viscosity and surface tension gradients, resulting from the redistribution of the surfactant [19]. Blawzdziewicz et al. [20] showed that the surfactant becomes incompressible under low-capillary-number conditions. The capillary number, 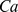, in this system can be defined as 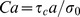, where 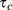 is the scale of the bulk stress, 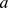 is the half body length, and 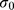 is the equilibrium surface tension. 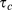 can be approximated by dividing the thrust force for swimming by the surface area of a cell, i.e, 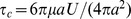, which becomes 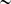
27 mPa by assuming 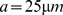 and the swimming velocity, U is 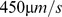. 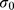 of Tween 20 with CMC is 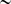
42 mN/m. The Ca in the present system can be estimated as 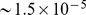, which is much smaller than unity, i.e. 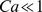. Thus, the surface mobility at the WAI is suppressed due to the surfactant incompressibility, as well as the surface shear viscosity in the present system.

**Figure 5:**
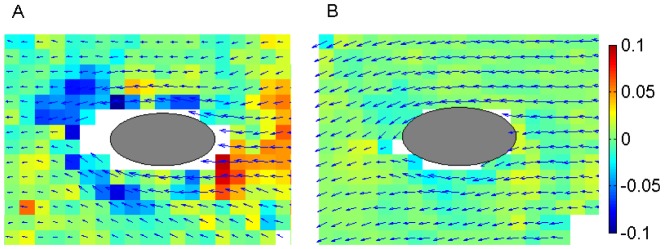
Velocity vectors surrounding a single cell swimming at the WAI. The color indicates the surface divergence of the surface velocity normalized by the swimming velocity of the cell. (A) Without a surfactant (B) With 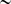
0.04 mM/m^3^ of surfactant.

To evaluate the difference in the flow field in the presence of a surfactant surrounding a cell, we measured the 2-D surface velocity, 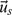, at the WAI and calculated its surface divergence 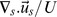 (Fig. 5) normalized by the swimming velocity of the cell, 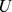. The flow field was measured using a particle tracking velocimeter, equipped with a confocal micro-PIV system [21]. The focal plane was set just below the WAI, and the trajectories of the 1-µm-diameter fluorescent beads were recorded at 100 fps. The velocity vectors were calculated by averaging the tracer velocities in each 7
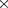
7-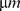 square box. The surface divergence was calculated numerically using the finite difference method.

Without surfactant, the cell swam with a low velocity, and the surrounding fluid was drawn from the anterior side, but pushed out from the posterior side, as shown in Fig. 5(A). The normalized surface divergence in the figure also showed that the surface velocity had a large divergence, which indicated three-dimensional (3-D) flow in the depth direction. We could not calculate the divergence in the white regions close to the cell body, because the velocity was too unsteady and particles disappeared quickly from the focal plane. However, we did observe a source at the anterior side, and a sink at the posterior side. The 3-D flow mixes the fluid at the WAI in the depth direction, as shown in Movie S3. With the addition of a surfactant (Fig. 5(B)), the cell swam with high velocity and did not appear to experience strong pushing or pulling forces as it moved through the fluid. Moreover, the normalized surface divergence in this case was small compared with that shown in Fig. 5(A). This is mainly because the surface mobility at the WAI was suppressed, due to the surfactant incompressibility as well as the surface shear viscosity. We can conclude that the entrapment of *Tetrahymena* is strongly affected by the physical surface properties and that the stress-free condition of the WAI without surfactant is the main mechanism of the entrapment phenomenon.

## Discussion

Ishikawa et. al. (2006) [22] derived basic equations for the lubrication theory of a sphere with a surface tangential velocity, i.e., a squirmer. In the present cell model, the surface velocity is generated on a force density shell, which has mainly a tangential velocity component, due to the no-slip boundary condition on the cell body. A squirmer is thus a good simplified model for understanding the hydrodynamics of the model cell near the interface. The results of Ishikawa et al. (2006) [22] showed that the torque exerted by the squirmer was proportional to the velocity difference, 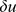, between the interface and the squirmer surface. The leading order term in the lubrication theory is 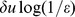, where 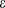 is the gap between two adjacent surfaces. To simplify the discussion, let the squirmer be placed parallel to the interface, without translational or rotational velocity. When the interface satisfies the no-slip condition, such as solid wall, the velocity of the squirmer surface is 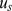, where 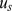 is the squirming velocity in the lubrication region, and the velocity at the solid wall is 0. Thus, 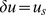, and the leading order term of the exerted torque is proportional to 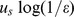. The squirmer tends to orient away from the solid wall, due to the torque. In this case, the rotational velocity is again proportional to 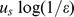. This is the main mechanism that causes the model cell to swim away from the solid wall. In contrast, when the interface satisfies the stress-free condition, the tangential component of the velocity at the interface has no gradient in the normal direction. Thus, the velocity becomes 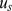 both at the squirmer surface and at the stress-free interface, i.e., 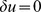, and the leading order term in the lubrication theory disappears. Because the next order effect comes not only from the lubrication region, but also from the whole cell body, the hydrodynamics in this case becomes qualitatively different from that with the no-slip surface, and a weaker torque is applied to the squirmer. This is the main reason why the model cell behaves differently at the no-slip and the stress-free interfaces. The tangential stress at the interface would also explain how cells at a water-oil interface behave in a similar manner as those at the WAI without a surfactant. The interfacial tension of the water-oil interface was indeed 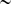
26 mN/m, that is, 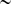
16 mN/m lower than the threshold value at which no cells could be found at the WAI.

The cells at the WAI tended to mix the surrounding fluid considerably (cf. Movie S3 and Fig. 5(A)). The trapped cells did not stop the ciliary beat (see Movie S4). This and Movie S3, in which we can see beads flow on top of the cells, imply that the fluid flow relative to the cell body was generated as usual. The cells, however, swam very slowly, which generated jet flows in the depth direction. Because the jet flow is downward, the surface velocity looks like a sink, as shown in Fig. 5(A). Cells change their position and orientation over time; thus, the jet flows generate chaotic advection flow, not only at the interface, but also in the depth direction. This suggests that the entrapment of cells enhances mass transport at the interface, which would likely affect the cells' metabolism. We note that similar entrapment phenomena at the WAI were also observed for *Volvox* (cf. Movie S2). The observed phenomena could be universal for ciliates. The results obtained from this study should be useful for a better understanding of the physiology of microorganisms at interfaces in nature and in industry.

## Materials and Methods

The cells used in the experiment were *Tetrahymena thermophila* (wild type: CH1). They were cultivated and observed in PYD at 27°C. The PYD was composed of 1% protease peptone, 0.5% yeast extract and 0.87% glucose in pure water. The cells were observed after two consecutive inocula. The first inoculum was performed by adding 20 µL of stock cells into 8 mL of fresh medium in a 60-mm-diameter circular Petri dish that was 15 mm in depth: 6 to 7 days after inoculum, 20 µL of these cells were added to 8 mL of fresh medium in the Petri dish.

The observations were made using an upright fluorescent microscope (Leica, DM 4000B, German) with a 10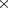 objective lens (Leica, HCX PL FLUOTAR 10×/0.30 PH1) and a CMOS camera (Basler A602fc, German). To record the cell motion at the interface, as well as inside the medium we proceeded as follows: The medium with cells was put into a circular PDMS pool that was 25 mm in diameter and 2 mm in depth. To avoid evaporation, we obtained 100% humidity conditions by enclosing the pool in a 60-mm-diameter Petri dish. Creating the enclosed space also prevented air flow from disturbing the cell motion at the WAI. The room in which the experiments were performed was maintained at 25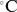 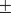
2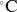.

The total concentration of the cells and the surface concentration were calculated using two different methods. The cell concentration in the medium was measured using plankton counting plates (Matsunami, Japan). First, the cells were fixed by adding formaldehyde, and then they were agitated and poured into counting plates. The advantage of this method was the ability to stop the motion of the cells without lysis. To calculate the surface concentration, on the other hand, fresh cells were poured into the PDMS pool, and after 10 minutes, five pictures of the cells at the WAI were taken. The pictures were taken at different locations in the pool, separated by at least 1 mm. This was repeated for six different samples, i.e., six different inocula. The error bars represent the standard deviation for the six values obtained.

To measure the swimming velocity inside the medium, swimming motions of cells in a plane at least 500 µm away from any interface were recorded. The motion of the cells at the WAI was recorded at least 2 mm from the walls of the pool. The trajectories of the cells were extracted by the plugin MJTrack of the software ImageJ (http://rsbweb.nuh.gov), and then the velocities of the cells were calculated. This was performed for six different inocula. In each inoculum, 20 cells were considered, giving a total of 120 values of velocity. The velocity of each cell was calculated by taking the average of 10 instantaneous velocities from 11 successive images of its track. The error bars shown on the graph of the velocity represent the standard deviation.

To devise the dimensions of the pool used in the series of experiments, we tried to define a diameter big enough to have an interface as flat as possible at its center, and a pool shallow enough to avoid bioconvection. We observed in preliminary studies that *Tetrahymena* became trapped by air bubbles inside the medium. The effect of the radius of curvature seems small, but it should be investigated in detail in future studies. Additionally, we did not observe a clear correlation between the bioconvection pattern and the repartition and motion of cells at the interface; however further investigation should be conducted in future studies.

## Supporting Information

Movie S1
**Example of Tetrahymena trapped at the water-air interface.** The movie was taken using the upright microscope (Leica, DM, German) with the 10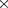 objective lens (Leica, HCX PL FLUOTAR 10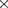/0.30 PH1) at 22 fps using dark field imaging. The display frame rate is also 22 fps. In this movie we can observe some trapped cells at the interface. They are moving in two-dimension and all of them are focused. The other cells are swimming in three-dimension. We can also observe some cells getting trapped at the interface, which slow them down greatly (e.g. top left corner at 3.77 s), and some others escaping from the interface, which makes them accelerate (e.g. middle right corner at 2.77 s).(AVI)

Movie S2
**Example of Volvox trapped at the water-air interface.** The movie was taken using the upright microscope (Olympus, UPlanSApo, Japan) with the 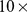 objective lens (Olympus, UPlanSApo, Japan) and the camera (Photron, FASTCAM SA3, USA) at 30 fps using bright field. The display frame rate is also 30 fps.(AVI)

Movie S3
**Example of Tetrahymena swimming close to a solid surface.** The movie was taken using the upright microscope (Leica, DM, German) with the 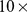 objective lens (Leica, HCX PL FLUOTAR 10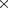/0.30 PH1) at 22 fps using phase contrast imaging. The display frame rate is also 22 fps. In this movie we can observe that the cells are easily swimming away from the solid surface.(AVI)

Movie S4
**Mixing induced by the trapped cells.** The movie was taken using the upright microscope(Leica DM 4000B, German) with the 10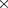 objective lens (Leica, HCX PL FLUOTAR 10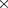/0.30 PH1) at 22 fps using dark field imaging. The display frame rate is also 22 fps. In this movie, we can observe the cell at the water-air interface as well as the 1 μm beads moving around the cells. We see the strong mixing induced by the entrapment of the cells.(AVI)
